# Circadian rhythmicity in prepulse inhibition of the acoustic startle response: A study of chronotype and time-of-day effects in young healthy adults

**DOI:** 10.1177/02698811251337397

**Published:** 2025-05-15

**Authors:** Satyam Chauhan, Ulrich Ettinger, Kaja Fassbender, Ray Norbury, Veena Kumari

**Affiliations:** 1 Department of Psychology, College of Health, Medicine and Life Sciences, Brunel University of London, Uxbridge, UK; 2 Centre for Cognitive and Clinical Neuroscience, College of Health, Medicine and Life Sciences, Brunel University of London, Uxbridge, UK; 3 Department of Psychology, University of Bonn, Bonn, Germany

**Keywords:** Chronotype, morningness-eveningness, sleep, habituation, prepulse inhibition, synchrony, time of day

## Abstract

**Background:**

Prepulse inhibition (PPI) of the acoustically elicited startle response is a widely used cross-species measure of sensorimotor gating. It is known to be reduced in various psychiatric disorders. Given previous reports of (a) disrupted PPI in young adults following overnight sleep deprivation and (b) disrupted sleep–wake cycles and psychiatric disorders being more common in evening than morning chronotypes, it is possible that there are chronobiological influences on human PPI.

**Aims:**

We investigated chronotype, time of day (ToD) and synchrony effects (i.e. optimal functioning at preferred ToD) in acoustic PPI in young healthy adults.

**Methods:**

Thirty-six adults, selected from a larger pool (*N* = 213) to represent morning, intermediate or evening chronotypes, were assessed on PPI (prepulse-to-pulse intervals: 30, 60 and 120-ms) on two occasions, 1 week apart: once in the morning (8:00–10:00) and once during the late afternoon (16:00–18:00).

**Results:**

There were no chronotype or synchrony effects on PPI. In the late afternoon, compared to the morning session, (i) there was greater startle amplitude on pulse-alone trials in association with higher schizotypy and (ii) greater PPI on 120-ms (but not 30-ms or 60-ms) PPI trials, but this effect became non-significant after covarying for schizotypy.

**Conclusions:**

Our findings showed no chronotype or synchrony effect on PPI, and offer further support for PPI to be a stable biomarker that is not significantly modulated by chronotype or ToD in healthy adults. ToD, however, may influence some startle parameters in association with schizotypy and should be considered in future studies of schizotypy and related populations.

## Introduction

Circadian rhythms cause considerable inter-individual differences in various mechanisms, including sleeping patterns and alertness/arousal levels, collectively known as chronotype (Adan et al., 2012). Chronotype is a multidimensional construct (Chauhan et al., 2023) that classifies individuals as ‘*morning chronotypes*’ (MCs; i.e., circadian peak arousal in the morning), ‘*evening chronotypes*’ (ECs; i.e., circadian peak arousal in the evening) or ‘*intermediate chronotypes*’ (ICs; i.e., no fixed circadian peak arousal). Given these inter-individual differences, it is possible to expect some variation in cognitive performance, including on tasks assessing attention and inhibition, in association with chronotype and/or time of day (ToD; Schmidt et al., 2007). When an individual’s performance is synchronised with their circadian arousal peak (May et al., 2023), it may result in a synchrony effect, i.e., superior performance at optimal ToD. There is evidence of chronobiological influences in performance on cognitive tasks requiring controlled processing of information (e.g. Lara et al., 2014; Martínez-Pérez et al., 2020; Rabi et al., 2022), although not consistently so (Schmidt et al., 2015; Song et al., 2018). To our knowledge, there are no studies examining chronotype or synchrony effects on human sensorimotor gating, as assessed by prepulse inhibition (PPI) of the startle response (Graham, 1975).

PPI of the startle response is a widely used cross-species measure of sensorimotor gating in healthy and clinical populations (reviews, Braff et al., 2001; Geyer and Braff, 1987; Santos-Carrasco and De la Casa, 2023; Swerdlow et al., 2016). It refers to a reliable reduction in startle response to a strong sensory stimulus (i.e. pulse) when preceded briefly (by 30–500 ms) by a weaker subthreshold stimulus (i.e. prepulse; Graham, 1975). Reduced PPI has been found in various psychiatric and neurodevelopmental disorders (Santos-Carrasco and De la Casa, 2023). Overnight sleep deprivation (SD) has been reported to disrupt PPI when young healthy participants are tested in the morning following overnight SD (Meyhöfer et al., 2019; Petrovsky et al., 2014). Interestingly, no PPI disruption was seen in a recent study where participants were tested in the evening following a 36-hour SD (Vizeli et al., 2023). Furthermore, in one study of female rats (Adam et al., 2008), ToD was reported to influence PPI selectively with intense 86-dB prepulses (no effect on 74–82 dB prepulses) with lower PPI in the morning (light phase) relative to the evening (dark phase).

No study has yet investigated chronobiological influences on human PPI. Given previous reports of disrupted sleep–wake cycles being more common in ECs than MCs (Chauhan et al., 2024a, 2024b; Muzni et al., 2021), it is possible that there are chronobiological influences on human PPI. This is an important area of enquiry since the PPI model has been widely utilised not only to study various human psychopathologies (San-Martin et al., 2020; Sun et al., 2024) but also to discover potential new treatments for schizophrenia (Geyer, 2006; Light and Swerdlow, 2020).

The primary aim of this study, therefore, was to investigate, for the first time, the effects of chronotype, ToD, and synchrony on PPI of the acoustic startle response in young healthy adults, as well as any associations between PPI and sleep quality over the past week. We tentatively hypothesised greater PPI at optimal ToD in all chronotypes (i.e., synchrony effect), based on evidence of such effects in some cognitive tasks (executive function) that show a positive association with PPI (e.g., Giakoumaki et al., 2008; Kumari et al., 2007) and a negative association between morning PPI and poor sleep quality, given previous reports of PPI disruption following SD when tested in the morning (Meyhöfer et al., 2019; Petrovsky et al., 2014). A secondary aim, given previous reports of a negative association between PPI and schizotypy (Giakoumaki, 2012; Giakoumaki et al., 2020) and impulsivity (Gee et al., 2015), was to explore possible associations between psychometric measures of schizotypy and impulsivity and PPI in the morning and late afternoon assessments, expecting the same pattern of associations in both sessions.

## Methods

### Participants and design

The study involved 45 young healthy adults. These participants were selected from a larger pool of adults who had completed the Morningness-Eveningness Questionnaire (MEQ; Horne and Ostberg, 1976) for a previous study (Chauhan et al., 2024b). Of 56 MCs (MEQ scores: 54–86), 83 ICs (MEQ scores: 42–53), and 46 ECs (MEQ scores: 16–41) in this larger pool, we invited 20 adults per chronotype group (providing 85% power at *p* < 0.05 with an a priori effect size of *f* = 0.4; Faul et al., 2007) who also met the study inclusion criteria to participate. The general study inclusion criteria for participation were as follows: (i) age between 18 and 40 years, (ii) resident in the UK, (iii) native/proficient English speaker, (iv) no hearing impairment, and (v) no current diagnosis of any mental disorders or drug abuse. In addition, to minimise sex differences and menstrual cycle-related influences (in women) in PPI (reviews, Hantsoo et al., 2018; Kumari, 2011; Santos-Carrasco and De la Casa, 2024), we invited only those females who were taking hormonal contraceptives (Naysmith et al., 2022). Of 20 participants per chronotype group invited to take part, 14 MCs, 17 ICs, and 14 ECs participated.

All participants took part in two identical sessions, 1 week apart: once in the morning between 8:00-10:00 hour and once in the late afternoon between 16:00-18:00 hour. Of the initial 45 participants, 9 participants were excluded due to noise/artifact contamination in startle assessments in one or both sessions, leaving a final sample of 36 participants (8 MCs, 15 ICs, and 13 ECs). Of these 36, 19 participants attended the morning session first, and the remaining 17 participants attended the late afternoon session first.

The study was approved by the College of Health, Medicine and Life Science Research Ethics Committee, Brunel University of London (ref no. 36745-A-Jan/2023-43031-3). All participants signed a consent form and, upon completion, were compensated with a £20 Amazon gift voucher.

### Self-report measures

During the screening session, all participants completed self-report measures of chronotype, sleep quality (over the past month), schizotypy and impulsivity. In addition, all participants completed the self-report measure of sleep quality (over the past week) prior to both PPI sessions.

#### Chronotype

The MEQ (Horne and Ostberg, 1976) was used to assess chronotype. It is a 19-item self-report measure with high reliability (*a* = 0.83, Horne and Ostberg, 1976; in the current sample, *a* = 0.87). Of the 19 items, 12 have a *Likert scale* (each item presenting four options with the lowest values reflecting preference for eveningness), and 7 have a *time scale* (each item is divided into periods of 15 min spanning a time frame of 7 hour). All responses are tallied to obtain a global score (range: 16–86), with higher scores indicating higher morningness.

#### Sleep quality

The *Pittsburgh Sleep Quality Index* (PSQI; Buysse et al., 1989) was used to assess sleep quality. It is a 19-item self-report measure with high internal consistency (*a* = 0.83, Buysse et al., 1989; current sample, screening session: *a* = 0.71, morning session, *a* = 0.74, late afternoon session *a* = 0.73) and assesses different sleep facets (i.e. daytime dysfunction, medication use, sleep disturbances, sleep efficiency, sleep duration, sleep latency, sleep quality). Participants answer each item by relating it to their past month’s experience (Buysse et al., 1989); each item is then tallied up to yield a global score (range: 0–21). Prior to both PPI sessions, the PSQI was administered with a slight modification, that is, to assess sleep quality over the past week. Higher scores indicate poor sleep quality.

#### Schizotypy

The *Oxford-Liverpool Inventory of Feelings and Emotions* (s-OLIFE; Mason et al., 2005) was used to assess schizotypy. It is a 43-item self-report measure with high reliability (*a* = 0.78–0.87, Fonseca-Pedrero et al., 2015). Each item belongs to one of the four subscales: *Unusual Experiences* (12 items), *Cognitive Disorganization* (11 items), *Introvertive Anhedonia* (10 items) and *Impulsive Non-conformity* (10 items). All items require a binary response (i.e., yes or no). Higher scores indicate higher levels of schizotypy. Cronbach’s alpha coefficients in the current sample for Unusual Experiences, Cognitive Disorganisation, Introvertive Anhedonia and Impulsive Nonconformity were 0.78, 0.83, 0.53 and 0.58, respectively.

#### Impulsivity

The *Impulsive Behaviour Scale*-*Short Version* (S-UPPS-P; Cyders et al., 2014) was used to assess impulsivity. It is a 20-item self-report measure with high reliability (total scale, *a* = 0.74–0.88; Cyders et al., 2014). It has five subscales (five items each): *Positive Urgency, Negative Urgency, Sensation Seeking, Lack of Perseverance* and *Lack of Premeditation*. Higher scores indicate higher levels of impulsivity. Cronbach’s alpha in the current sample for Positive Urgency, Negative Urgency, Sensation Seeking, Lack of Perseverance and Lack of Premeditation were 0.75, 0.72, 0.71, 0.59 and 0.78, respectively.

### PPI assessment: Startle paradigm and procedure

A commercially available human startle response monitoring system (SR-Lab, San Diego, California, USA) was used to generate and deliver the acoustic stimuli through headphones (binaurally) and record the electromyography (EMG) activity.

The session started with a 2-minute acclimatisation period during which all participants were exposed via headphones to 70-dB (A) continuous white noise. The pulse-alone stimulus was a 40-ms presentation of 114-dB (A) white noise, and the prepulse stimulus was a 20-ms presentation of 84-dB (A) white noise, both over 70-dB (A) continuous background noise (Kumari et al., 2024). In total, participants received 46 startle-eliciting stimuli. Of these 46 trials, the first five and the last five were the pulse-alone stimuli for measuring startle habituation. The remaining 36 trials were arranged in three blocks of twelve trials each. Each of the three blocks included: three pulse-alone trials, three PPI trials (PPI30) where the prepulse (onset) to pulse (onset) interval was 30-ms, three PPI trials (PPI60) where the prepulse (onset) to pulse (onset) interval was 60-ms, and three PPI trials (PPI120) where the prepulse (onset) to pulse (onset) interval was 120-ms. Within each block, pulse-alone and PPI trials were ordered pseudo-randomly to avoid repetition of any particular trial type in a row.

The eye blink component of the startle response was measured by recording EMG activity of the orbicularis oculi muscle underneath the right eye by placing two miniature silver/silver chloride electrodes filled with Dracard electrolyte paste (SLE, Croydon, UK) and a ground electrode on the mastoid behind the right ear. The amplification gain control for the EMG signal was kept constant for all participants and all sessions. During the testing session, participants were seated comfortably in a chair. All participants were told that the study aimed to investigate their reactivity to various noises played through headphones at different ToD and that they should neither ignore nor try to attend these noises. They were requested to remain relaxed but stay awake with their eyes kept open throughout the experiment. Participants had been asked to refrain from smoking for 2 hours, given the widely reported influence of nicotine in PPI (Kumari et al., 1997; Kumari and Postma, 2005), and also from drinking caffeine for 3 hours, and consuming alcohol for 24 hours prior to their scheduled testing sessions.

Scoring criteria were identical to those reported by Kumari et al. (2023, 2024). Briefly stated, recorded EMG activity was band-pass filtered, as recommended by the SR-Lab. Analogue band-pass filtering occurred before digitising. The high-pass and low-pass cut-off frequencies were 50 and 1000 Hz, respectively. EMG data were processed off-line, blind to self-report data, using the analytic programme of the SR-Lab for response amplitude (in arbitrary analogue-to-digital (A/D) units; 1 unit = 2.62 μV). The scoring programme contained a rolling average routine, which smoothed the rectified EMG response. The onset of the startle response was defined by a shift of 10 A/D units from the baseline value occurring within 20–120 ms from the startle stimulus onset.

### Statistical analysis

All analyses were performed using the Statistical Package for Social Sciences (for Windows, version 29; IBM, New York, New York, USA). Alpha level for testing significance was maintained at *p* < 0.05 unless stated otherwise. The data properties of all measures were examined and found suitable for parametric data analysis methods.

#### Sample characteristics

Possible group differences in age and various self-report measures, except sleep quality, were explored using one-way analysis of variance (ANOVA) with chronotype as a between-subject factor, followed by post hoc mean comparisons as appropriate. Group differences in sleep quality were assessed using a 3 (Group: MCs, ICs, ECs) × 3 (Session: screening, morning, late afternoon) ANOVA with Group as a between-subjects factor, and Session as a within-subjects factor, followed by lower-order ANOVAs and post hoc mean comparisons as appropriate. Prior to these analyses, we explored sex differences in various self-report measures across the three groups (*n* too small for including both Group and Sex factors in the same ANOVA) but found no significant sex difference in any measure (thus, Sex was not considered further as a factor).

#### Chronotype and ToD influences in startle measures

**Figure 1.**
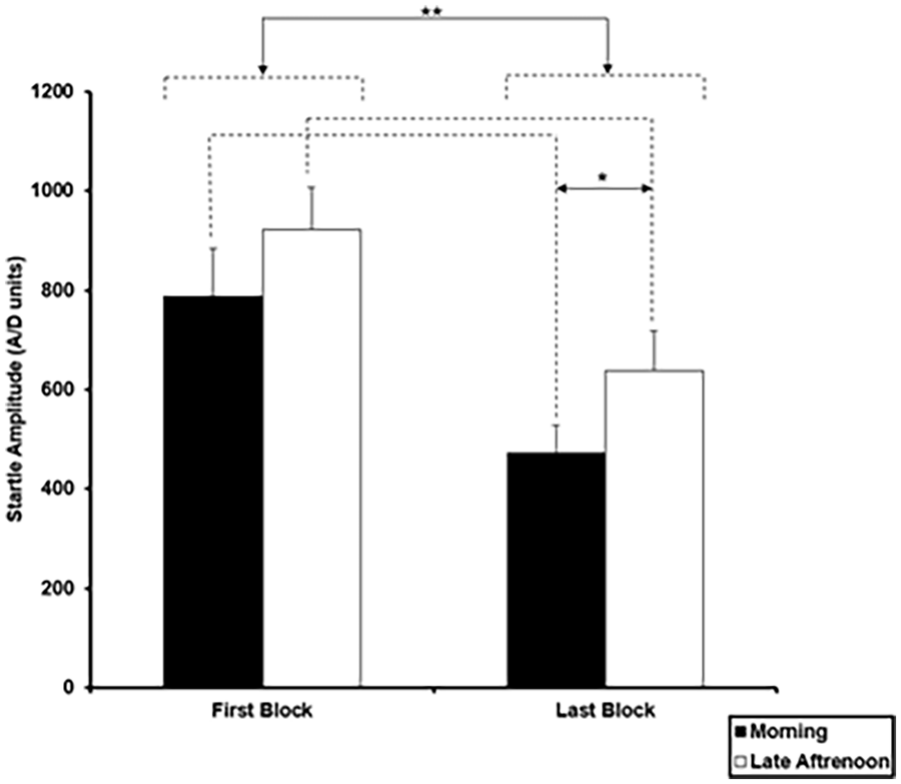
Mean startle amplitude in analogue-to-digit (A/D) units on the first and last block of pulse-alone trials (5 trials each) in the morning and late afternoon session (*p = 0.05; **p = 0.01). Error bars represent +1 standard error of the mean (SEM).

PPI was calculated as ([a − b]/a)] × 100, where ‘a’ = pulse-alone amplitude (mean amplitude response on nine pulse-alone trials during the three middle blocks; see Figure 1) and ‘b’ = amplitude over PPI trials. Before examining possible Chronotype and ToD effects on PPI, we analysed average startle amplitudes on the first and last block of five pulse-alone trials using a 3 (Group: MCs, ICs, ECs) × 2 (ToD: morning, late afternoon) × 2 (first block, last block) ANOVA, with Group as a between-subjects factor, and ToD and Block as within-subjects factors, followed by lower-order ANOVAs and post hoc mean comparisons as appropriate. Given a significant Group (chronotype) effect in schizotypy (see Results), we re-evaluated all effects involving Group, ToD or Block factors in pulse-alone amplitudes after covarying for schizotypy (s-OLIFE total score).

Habituation was also calculated (% reduction in average amplitude from the first block of five pulse-alone trials to the last block of five pulse-alone trials) and analysed using a 2 (Group) × 2 (ToD) ANOVA, with repeated measures on ToD, with and without covarying for schizotypy.

PPI scores were examined using a 3 (Group: MCs, ICs, ECs) × 2 (ToD: morning, late afternoon) × 3 (PPI Trial Type: 30-ms, 60-ms, 120-ms) ANOVA, with Group as a between-subjects factor, and ToD and PPI Trial Type as within-subjects factors, followed by lower-order ANOVAs and post hoc mean comparisons as appropriate. Given a significant ToD effect on amplitude on the first and last block of five pulse-alone trials (see Results), a 3 (Group) × 2 (ToD) ANOVA was also run-on mean startle amplitude on pulse-alone trials that were presented mixed with the PPI trials, and any significant effects from ANOVA on PPI scores were re-evaluated after covarying for mean amplitude on these pulse-alone trials, and also covarying for schizotypy. Lastly, we conducted 3 (Group) × 2 (ToD) × 4 (Trial Type: pulse-alone, PPI 30-ms, PPI 60-ms, PPI 120-ms) ANOVA on latencies to startle peak, with Group as a between-subjects factor, and ToD and PPI Trial Type as within-subjects factors, with and without covarying for schizotypy. Significant main effects and interactions were followed by lower-order ANOVAs and post hoc mean comparisons as appropriate.

Prior to these analyses, Sex (male, female) and Experimental Order (morning first, late afternoon first) were examined (separately to maximise power) as additional between-group factors in all ANOVAs and not found to have any main or interactive effects in any of the startle measures (thus not considered further). The assumption of sphericity was assessed using Mauchly’s test in all ANOVAs for factors involving a repeated measure. If the assumption of sphericity was found to be violated, the Greenhouse–Geisser correction was applied. Effect sizes, when reported, are partial eta squared (ηp^2^; the proportion of variance associated with a factor).

#### Self-report and startle measures: Inter-relationships

Correlational analyses (Pearson’s *r*) were used to examine the relationship of sleep quality (global scores), schizotypy and impulsivity with startle measures. Significant correlations (*p* < 0.05) that had not been hypothesised a priori were re-evaluated after applying Bonferroni correction to control family-wise Type 1 error.

## Results

### Sample characteristics

Demographic characteristics are presented in Table 1. There was a main effect of Group for total schizotypy (*F*_(2, 33)_ = 6.70, *p* = 0.004, ηp^2^ = 0.289; MCs scoring lower than ICs (*p* < 0.001) and ECs (*p* = 0.028)); schizotypy subscales, *Cognitive Disorganisation* (*F*_(2, 33)_ = 3.93, *p* = 0.029, ηp^2^ = 0.192; MCs scoring lower than ICs (*p* = 0.009)), *Introvertive Anhedonia* (*F*_(2, 33)_ = 5.43, *p* = 0.009, ηp^2^ = 0.248; MCs scoring lower than ICs (*p* = 0.003) and ECs (*p* = 0.015)), *Impulsive Nonconformity* (*F*_(2, 33)_ = 4.98, *p* = 0.013, ηp^2^ = 0.232; MCs scoring lower than ICs (*p* = 0.003) and ECs (*p* = 0.040)). No other personality measure showed a significant group difference (*p* > 0.05). For sleep quality, no main effect of Group (*F*_(2, 31)_ = 0.866, *p* = 0.430, ηp^2^ = 0.053), Session (*F*_(2, 62)_ = 1.05, *p* = 0.353, ηp^2^ = 0.033) or a Group × Session interaction (*F*_(4, 62)_ = 1.69, *p* = 0.164, ηp^2^ = 0.098) was found.

**Table 1.** Sample characterisation measures.

Variables	Morning types (*n* = 8, 3M/5F)		Intermediate types (*n* = 15, 8M, 7F)		Evening types (*n* = 13, 7M/6F)		Total (*n* = 36, 18M/18F)	
	Mean ± SD	Range	Mean	Range	Mean ± SD	Range	Mean ± SD	Range
Age	27.5 ± 4.78	21–36	22.73 ± 2.46	18–27	27.08 ± 6.18	18–39	25.36 ± 5.02	18–39
Chronotype								
MEQ	58.5 ± 4.62	54–67	48.20 ± 3.98	42–53	34.30 ± 6.26	21–41	45.47 ± 10.60	21–67
Sleep quality								
PSQI (S)	4.75 ± 2.31	2–8	6.33 ± 2.52	1–10	7.15 ± 1.90	4–10	6.27 ± 2.38	1–10
PSQI (M)	6.25 ± 3.49	3–13	6.06 ± 3.15	3–13	6.91 ± 3.31	2–13	6.4 ± 3.21	2–13
PSQI (E)	4.50 ± 3.46	1–11	6.40 ± 2.92	2–13	6.41 ± 0.60	3–11	5.97 ± 2.97	1–13
Schizotypy								
UE	3.5 ± 2.92	0–8	5.73 ± 3.15	0–11	4.61 ± 2.95	0–10	4.83 ± 3.07	0–11
CD	4.25 ± 3.19	1–9	7.93 ± 2.73	3–11	6.38 ± 3.20	1–11	6.55 ± 3.25	1–11
IA	1.62 ± 1.18	0–4	4.26 ± 1.98	1–8	3.76 ± 2.04	0–7	3.5 ± 2.09	0–8
IN	1.12 ± 0.83	0–2	3.6 ± 2.06	0–8	2.84 ± 1.86	0–6	2.77 ± 1.98	0–8
Total score	10.50 ± 6.80	5–22	21.53 ± 6.67	9–29	17.61 ± 7.17	5–29	17.66 ± 7.92	5–29
Impulsivity								
PU	7 ± 2.26	4–11	9.26 ± 3.30	4–16	8.76 ± 2.97	4–15	8.58 ± 3.03	4–16
NU	7.37 ± 2.06	5–11	9.46 ± 3.11	5–16	9.61 ± 2.81	5–16	9.05 ± 2.87	5–16
SS	11.25 ± 2.81	5–14	11.6 ± 2.19	8–15	10.53 ± 3.66	5–16	11.13 ± 2.88	5–16
LP	6.75 ± 1.98	4–9	7.53 ± 1.76	5–11	7.07 ± 2.10	4–11	7.19 ± 1.90	4–11
LPre	7.5 ± 2.87	4–11	7.33 ± 2.05	5–11	6.84 ± 2.07	5–10	7.19 ± 2.21	4–11

MEQ: Morningness-Eveningness Questionnaire; PSQI (M): Pittsburgh sleep quality index (evening); PSQI (E): Pittsburgh sleep quality index (morning); PSQI (S): Pittsburgh sleep quality index (screening); UE: unusual experiences; CD: cognitive disorganisation; IA: introvertive anhedonia; IN: impulsive nonconformity; PU: positive urgency; NU: negative urgency; SS: sensation seeking; LP: lack of perseverance; LPre: lack of premeditation.

### Chronotype and ToD influences in startle amplitude, habituation and PPI

Group × ToD × Block ANOVA on startle amplitude over the first and last block of pulse alone trials showed significant main effects of Block (*F*_(1, 33)_ = 53.48, *p* < 0.001, ηp^2^ = 0.618) indicating higher amplitude on the first block (824.34 ± 84.52), compared to the last block of trials (523.64 ± 64.12), and also of ToD (*F*_(1, 33)_ = 6.41, *p* = 0.016, ηp^2^ = 0.163) indicating generally lower amplitudes in the morning (601.22 ± 73.03) than in the late afternoon (746.17 ± 81.92) (Figure 1); there was no interaction involving Group, Block or ToD factors (*p* > 0.05). After covarying for schizotypy, the main effect of Block remained significant (*F*_(1, 32)_ = 16.54, *p* < 0.001, ηp^2^ = 0.341), the main effect of ToD became non-significant (*F*_(1, 32)_ = 1.29, *p* = 0.26, ηp^2^ = 0.23), and there was a significant ToD × Schizotypy interaction (*F*_(1, 32)_ = 5.49, *p* = 0.025, ηp^2^ = 0.146) explained by a trend for greater increase in pulse-alone amplitude from the morning to the late afternoon session in association with higher schizotypy (across the first and last blocks, *r* = 0.309, *p* = 0.067; first block, *r* = 0.289, *p* = 0.087; last block, *r* = 0.196, *p* = 0.253).

Group × ToD ANOVA on habituation scores (% reduction in amplitude from the first to the last block of pulse-alone trials) also demonstrated no effects of Group (*F*_(2, 33)_ = 2.58, *p* = 0.09, ηp^2^ = 0.135), ToD (*F*_(1, 33)_ = 0.031, *p* = 0.862, ηp^2^ = 0.001) or Group × ToD interaction (*F*_(2, 32)_ = 0.308, *p* = 0.737, ηp^2^ = 0.018). The same pattern of effects was observed after covarying for schizotypy.

The Group × ToD ANOVA on amplitude over pulse-alone trials that were presented interspersed with the PPI trials revealed no effect of Group (*F*_(2, 33)_ = 1.98, *p* = 0.153, ηp^2^ = 0.108), ToD (*F*_(1, 33)_ = 1.34, *p* = 0.255, ηp^2^ = 0.039) or a Group × ToD interaction (*F*_(2, 33)_ = 0.06, *p* = 0.939, ηp^2^ = 0.004). After covarying for schizotypy, Group, ToD and Group × ToD effects remained non-significant but a significant ToD × Schizotypy interaction emerged (*F*_(2, 32)_ = 6.19, *p* = 0.018, ηp^2^ = 0.162); this ToD × Schizotypy interaction was explained by a greater increase in pulse-alone amplitude from the morning to the late afternoon session in association with higher schizotypy (*r* = 0.335, *p* = 0.046), as noted earlier also for average amplitude on the first and the last block of pulse-alone trials.

**Figure 2.**
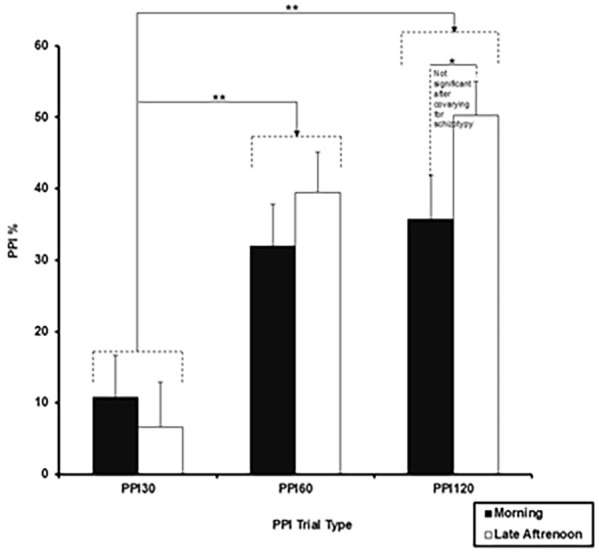
Prepulse inhibition (PPI) with 30-, 60- and 120-ms prepulse-to-pulse interval trials in the morning and late afternoon sessions (*p = 0.05; **p = 0.01). Error bars represent +1 SEM.

Group × ToD × PPI Trial Type ANOVA on PPI scores showed a significant main effect of Trial Type (*F*_(2, 66)_ = 29.12, *p* < 0.001, ηp^2^ = 0.469), with a linear increase in PPI from 30-ms through 60-ms to 120-ms PPI trials (linear *F*_(1, 33)_ = 28.38, *p* < 0.001, ηp^2^ = 0.462). There was also a significant PPI Trial Type × ToD interaction (*F*_(1.70, 56.06)_ = 3.88, *p* = 0.03, ηp^2^ = 0.105), explained by significantly lower PPI on 120-ms PPI trials in the morning session, compared to the late afternoon session (*t*_35_ = 2.25, *p* = 0.015) (Figure 2); there was no significant ToD-related difference in PPI on 60-ms (*t*_35_ = 1.18, *p* = 0.122) or 30-ms (*t*_35_ = 0.81 *p* = 0.210) trials. There was no main effect or any interaction involving Group (all *p-*values > 0.05). The ToD × Trial Type interaction was marginally significant after co-varying for pulse-alone amplitude (*F*_(1.64, 51.03)_ = 3.49, *p* = 0.047, ηp^2^ = 0.101); it became non-significant after covarying for schizotypy (*F*_(1.70, 54.38)_ = 1.85, *p* = 0.173, ηp^2^ = 0.055) though there was no main effect or any interaction involving Schizotypy.

### Chronotype and ToD influence on startle latency

The Group × ToD × Trial Type ANOVA on startle latencies to peak revealed a significant main effect of ToD (*F*_(1, 33)_ = 9.53, *p* = 0.004, ηp^2^ = 0.224) with longer latencies, on average, in the late afternoon session (55 ± 1.17) compared to the morning session (49.33 ± 1.65) and also of Trial Type (*F*_(3, 99)_ = 34.23, *p* < 0.001, ηp^2^ = 0.509) with longer latencies (*p* < 0.001) on pulse-alone trials (60.35 ± 1.73) compared to all PPI trials (PPI 30-ms (50.74 ± 1.01), PPI 60-ms (48.30 ± 1.21), PPI 120-ms (49.29 ± 1.42)), and for PPI 30-ms trials compared to PPI-60 trials (*p* = 0.030). No other main or interaction effects were significant. After covarying for schizotypy, the main effect of Trial Type remained significant (*F*_(3, 96)_ = 9.46, *p* < 0.001, ηp^2^ = 0.228) but there was no effect of ToD (*F*_(1, 32)_ = 0.25, *p* = 0.621, ηp^2^ = 0.008) and no main effect or any interaction involving Schizotypy.

### Self-report and startle measures: Inter-relationships

Of the hypothesised correlations, only Positive Urgency was associated with less than 30-ms PPI30 (*r* = −0.354, *p* = 0.034) and 60-ms PPI60 (*r* = −0.372, *p* = 0.026) in the morning session (Table 2). Sleep quality (over the past week) was not significantly associated with any startle measures (see Table 2). In exploratory correlational analyses, higher morningness did not show significant association with any startle measure except a negative correlation with amplitude on the last block of pulse-alone trials in the late afternoon session (*r* = −0.344, *p* = 0.040); Sensation Seeking was associated with higher amplitude on the first block of pulse-alone trials (*r* = 0.354, *p* = 0.037), more habituation (*r* = −0.375, *p* = 0.026) and longer PPI120 startle latencies to peak (*r* = 0.392, *p* = 0.018) in the morning session (Table 2); Impulsive Nonconformity was associated with higher amplitude on pulse-alone trials (*r*-values 0.228 to −0.471) and a weaker habituation from the first block to the last block of pulse-alone trials (*r* = −0.336, *p* = 0.045) in the late afternoon session (Table 2); and Negative Urgency was associated with shorter PPI60 startle latencies to peak (*r* = −0.426, *p* = 0.10). None of these exploratory correlations were strong enough to survive a correction for multiple correlations (and thus not discussed further). Lastly, startle variables (pulse-alone amplitude, PPI) from the morning and late afternoon sessions were generally positively correlated across the entire sample (see Supplemental Table 1).

**Table 2.** Self-report and startle measures: Inter-relationships.

Variables	CT	PSQI		s-OLIFE				UPPS-P				
	MEQ	M	LA	UE	CD	IA	IN	PU	NU	SS	LP	LPre
Morning session	*r* (*p*)	*r* (*p*)	*r* (*p*)	*r* (*p*)	*r* (*p*)	*r* (*p*)	*r* (*p*)	*r* (*p*)	*r* (*p*)	*r* (*p*)	*r* (*p*)	*r* (*p*)
Pulse-alone amplitude – first block	−0.184 (0.282)	−0.167 (0.339)	0.114 (0.516)	−0.277 (0.102)	−0.210 (0.219)	0.022 (0.897)	0.167 (0.331)	0.164 (0.340)	0.052 (0.764)	0.346^#^ (0.039)	0.061 (0.724)	−0.002 (0.992)
Pulse-alone amplitude – last block	−0.240 (0.159)	−0.109 (0.534)	0.119 (0.495)	−0.189 (0.270)	0.034 (0.842)	0.056 (0.746)	0.190 (0.266)	0.200 (0.241)	0.206 (0.227)	0.135 (0.432)	−0.121 (0.484)	−0.056 (0.744)
Habituation: Reduction from first to last block	0.101 (0.557)	−0.249 (0.149)	−0.134 (0.442)	0.008 (0.964)	−0.135 (0.432)	−0.009 (0.959)	−0.071 (0.891)	−0.131 (0.446)	−0.225 (0.188)	0.361^#^ (0.031)	0.214 (0.210)	0.089 (0.606)
Pulse-alone amplitude – PPI experiment	−0.228 (0.181)	−0.105 (0.548)	0.136 (0.435)	−0.161 (0.348)	0.030 (0.860)	0.045 (0.796)	0.204 (0.234)	0.192 (0.263)	0.169 (0.324)	0.166 (0.334)	0.024 (0.888)	−0.006 (0.971)
PPI30	−0.183 (0.286)	−0.053 (0.761)	0.085 (0.628)	−0.121 (0.482)	−0.055 (0.748)	0.273 (0.107)	−0.155 (0.366)	−0.354 (0.034)	−0.096 (0.579)	0.039 (0.822)	−0.132 (0.442)	−0.108 (0.530)
PPI60	−0.048 (0.782)	−0.093 (0.594)	0.022 (0.899)	−0.118 (0.494)	−0.104 (0.545)	0.183 (0.286)	−0.009 (0.959)	−0.372 (0.026)	0.045 (0.793)	−0.096 (0.576)	−0.035 (0.841)	−0.066 (0.703)
PPI120	−0.065 (0.706)	−0.105 (0.548)	0.214 (0.218)	0.171 (0.319)	0.144 (0.403)	0.217 (0.205)	0.124 (0.470)	−0.039 (0.821)	0.037 (0.832)	−0.023 (0.895)	0.018 (0.917)	−0.131 (0.448)
Late afternoon session	*r* (*p*)	*r* (*p*)	*r* (*p*)	*r* (*p*)	*r* (*p*)	*r* (*p*)	*r* (*p*)	*r* (*p*)	*r* (*p*)	*r* (*p*)	*r* (*p*)	*r* (*p*)
Pulse-alone amplitude – first block	−0.229 (0.179)	−0.101 (0.562)	0.164 (0.345)	0.042 (0.807)	0.128 (0.455)	0.012 (0.945)	0.228 (0.181)	0.231 (0.175)	0.149 (0.387)	0.114 (0.506)	0.025 (0.885)	−0.050 (0.770)
Pulse-alone amplitude – last block	−0.344^#^ (0.040)	−0.129 (0.461)	0.103 (0.556)	−0.126 (0.465)	0.143 (0.405)	−0.008 (0.963)	0.351^#^ (0.036)	0.283 (0.094)	0.229 (0.180)	0.276 (0.103)	0.011 (0.948)	0.025 (0.884)
Habituation: Reduction from first to last block	0.291 (0.086)	−0.004 (0.982)	−0.074 (0.673)	0.232 (0.174)	−0.121 (0.482)	−0.062 (0.721)	−0.336^#^ (0.045)	−0.139 (0.419)	−0.159 (0.356)	−0.199 (0.245)	−0.026 (0.880)	−0.084 (0.627)
Pulse-alone amplitude – PPI experiment	−0.262 (0.123)	−0.150 (0.390)	0.034 (0.846)	−0.044 (0.800)	0.258 (0.129)	−0.054 (0.754)	0.471^#^ (0.004)	0.274 (0.105)	0.311 (0.065)	0.062 (0.718)	0.094 (0.584)	0.037 (0.830)
PPI30	−0.048 (0.781)	−0.106 (0.544)	−0.059 (0.737)	−0.202 (0.237)	−0.164 (0.339)	−0.212 (0.215)	0.150 (0.382)	−0.097 (0.572)	0.086 (0.619)	0.196 (0.251)	−0.124 (0.469)	0.007 (0.968)
PPI60	0.047 (0.784)	−0.188 (0.279)	−0.130 (0.455)	−0.040 (0.817)	0.140 (0.416)	−0.141 (0.411)	0.312 (0.064)	−0.196 (0.252)	0.266 (0.116)	−0.177 (0.303)	0.116 (0.499)	0.031 (0.857)
PPI120	−0.278 (0.100)	−0.068 (0.696)	0.102 (0.560)	−0.058 (0.735)	−0.035 (0.840)	−0.113 (0.510)	0.236 (0.165)	−0.030 (0.861)	0.256 (0.132)	0.143 (0.405)	−0.054 (0.756)	−0.195 (0.255)

CT: chronotype; MEQ: Morningness-Eveningness Questionnaire; M: morning; LA: late-afternoon; PSQI: Pittsburgh sleep quality index; s-OLIFE: short Oxford-liverpool inventory of feelings experiences; UE: unusual experiences; CD: cognitive disorganisation; IA: introvertive anhedonia; IN: impulsive nonconformity; UPPS-P: impulsive behaviour scale-short version; PU: positive urgency; NU: negative urgency; SS: sensation seeking; LP: lack of perseverance; LPre: lack of premeditation.

# Exploratory correlations and not significant after correction for multiple correlations.

## Discussion

The primary aim of the present study was to investigate possible effects of chronotype, ToD and synchrony (chronotype in relation to optimal ToD) on PPI of the acoustic startle response in young healthy adults. The main findings indicated (i) no chronotype or synchrony effects on startle amplitude, habituation or PPI, (ii) higher startle amplitude on pulse-alone trials, especially in association with a higher level of schizotypy (with schizotypy being lower in MCs compared to other chronotypes), during the late afternoon session compared to the morning session, (iii) marginally greater PPI on 120-ms prepulse-to-pulse interval trials, and longer latencies on all startle trial types in the late afternoon session compared to the morning session but all these effects became non-significant after covarying for schizotypy, (iv) no association of sleep quality with startle amplitude, habituation or PPI and (v) medium-sized negative association between a psychometric measure of Positive Urgency (impulsivity) and PPI during the morning session (with weaker and non-significant negative association with late afternoon PPI).

The failure to observe chronotype or synchrony effects on PPI or any startle measure offers no support for the first tentative hypothesis. This finding, however, provides further empirical support for Yang et al. (2007), suggesting no chronotype effect on tasks where performance relies mainly on automatic processing and does not require conscious effort on the part of the participant. Some studies in rodents have shown higher startle amplitude and alterations in startle latency as well as PPI during the dark, relative to the light phase (Adams et al., 2008; Chabot and Taylor, 1992; Davis and Sollberger, 1971). Other studies report no effects of circadian time on habituation and PPI and attributed any effects (where found) to lighting conditions and sex-related influences (Weiss et al., 1999). Our findings cannot be directly compared to the findings of these rodent studies, as we tested our participants in the morning (8:00–10:00 hour) and late afternoon (16:00–18:00 hour) in laboratory conditions with natural light. Nonetheless, we did observe a significant ToD effect in startle amplitude (higher in the late afternoon) in association with a higher level of schizotypy, with schizotypy being significantly lower in MCs than ICs and ECs. Higher startle amplitude in the afternoon may be related to increased arousal levels during late afternoon sessions, which has been referred to as the ‘wakeful maintenance zone’ (WMZ; i.e., 2–3 hour window of increased alertness levels prior to melatonin secretion onset in the evening; Dijk et al., 1992) known to facilitate attentional network (McMahon et al., 2021). This effect may be particularly pronounced in individuals with higher schizotypy who are also known to generally exhibit hyperreactivity (i.e., heightened sensitivity to external stimuli) and sensory overload (Torrens et al., 2023), lower sensorimotor and sensory gating (Freedman et al., 2020; Giakoumaki et al., 2020; Wan et al., 2017), impaired attention, slower processing speed, and reduced latent inhibition (Ettinger et al., 2015; Kumari and Ettinger, 2010). Our observations of greater PPI on 120-ms prepulse-to-pulse intervals and generally longer latencies to startle peak in the late afternoon compared to the morning session, both of which disappeared after covarying for schizotypy, might also be explained by similar mechanisms.

In line with numerous previous studies in humans, we found an increase in PPI on 30-ms to 60-ms and 120-ms PPI trials; and observed generally shorter latencies on PPI trials, compared to the pulse-alone trials (e.g. Aasen et al., 2005; Kumari et al., 2005). In our study, PPI on 30-ms to 60-ms trials was not at all impacted by chronotype or ToD, with only a weak (at best) effect of ToD in 120-ms PPI that was abolished after co-varying for schizotypy. PPI with short-to-medium (30–60 ms) lead intervals mainly involves automatic processes, whereas PPI with longer lead intervals may, in addition to automatic stimulus detection, also involve controlled processes. For example, some PPI enhancement has been observed when participants are required to pay attention to the prepulses (Schell et al., 2000). In general, our findings, especially for 30-ms and 60-ms PPI, are consistent with previous studies (Abel et al., 1998; Freudenberg et al., 2022; Ludewig et al., 2002) demonstrating stability of PPI in healthy young adults and add further support to its utility as a biomarker to advance schizophrenia therapeutics (Geyer, 2006; Light and Swerdlow, 2020).

Our findings also did not reveal any relationship between sleep quality and PPI or any startle measures. Although two previous studies have demonstrated disrupted PPI in the morning following overnight SD (Meyhöfer et al., 2019; Petrovsky et al., 2014), SD and poor sleep quality are conceptually very different and affect cognitive performance differently. Whilst acute SD has been consistently shown to influence cognitive functions (e.g. inhibition, working memory; Krause et al., 2017; Kumari and Ettinger, 2020), poor sleep quality may or may not have similar effects in young healthy adults when tested between 11:00 and 15:00 hour (Zavecz et al., 2020). As discussed earlier, these wakeful maintenance hours may facilitate performance due to increased vigilance and arousal levels at this ToD (McMahon et al., 2021). Of note, our sample also consisted predominantly of good sleepers who are known to have higher melatonin secretion (Fatemeh et al., 2022). Lastly, as expected, we found a negative correlation between a measure of impulsivity and PPI (Gee et al., 2015), which was significant only for the morning session, and weaker and non-significant in the late afternoon session, possibly due to the WMZ-related influences described earlier. As there is no other study investigating ToD influences in association with PPI with psychopathology-related traits, further work is needed to explore this possibility. Lastly, schizotypy did not show a significant direct association with PPI in this study, although a marginal ToD effect in 120-ms PPI was abolished after covarying for schizotypy, and ECs did have higher schizotypy than MCs in line with our earlier findings in larger samples (Chauhan et al., 2024a, 2024b).

The present study had some limitations. First, we did not measure subjective or objective arousal levels. Second, we restricted our sample to 18–40 years to ensure chronotype stability in this age range (Roenneberg et al., 2007), but it also means that our findings cannot be generalised to those <18 and >40 years of age. Third, although with a total *N* of 36 participants, our sample size was large enough to examine ToD effects (power: 0.86), there was limited power to examine Chronotype × ToD interaction. Fourth, we did not collect information on specific hormonal profiles of the contraceptive used (Ivek et al., 2024) or the participant’s sexual orientation (Rahman et al., 2003), both of which may affect baseline PPI. Further studies with a larger sample and a comprehensive characterisation of study participants are needed to confirm our findings while accounting for these limitations.

To conclude, the present study showed no significant chronotype or synchrony effects on PPI. Furthermore, there was no significant association between PPI and sleep quality in our sample of young healthy adults who, on average, were fairly good sleepers. Taken together, our findings suggest that PPI, especially with short-to-medium prepulse-to-pulse intervals, is a stable biomarker and not significantly modulated by chronotype or ToD in healthy young adults. Given the influence of schizotypy in ToD-related modulation of some startle parameters in this study, we recommend that future studies in people with schizotypy and schizophrenia spectrum conditions should aim to control for, or at least report, the ToD at which study participants are tested.

## Supplemental Material

sj-docx-1-jop-10.1177_02698811251337397 – Supplemental material for Circadian rhythmicity in prepulse inhibition of the acoustic startle response: A study of chronotype and time-of-day effects in young healthy adults Supplemental material, sj-docx-1-jop-10.1177_02698811251337397 for Circadian rhythmicity in prepulse inhibition of the acoustic startle response: A study of chronotype and time-of-day effects in young healthy adults by Satyam Chauhan, Ulrich Ettinger, Kaja Fassbender, Ray Norbury and Veena Kumari in Journal of Psychopharmacology
