# Supplementary material for: Circadian rhythmicity in prepulse inhibition of the acoustic startle response: A study of chronotype and time-of-day effects in young healthy adults
Source: J Psychopharmacol. 2025 May 15;39(8):759–68. doi: 10.1177/02698811251337397 (PMC12287558; doi:10.1177/02698811251337397)
Supplement: sj-docx-1-jop-10.1177_02698811251337397 – Supplemental material for Circadian rhythmicity in prepulse inhibition of the acoustic startle response: A study of chronotype and time-of-day effects in young healthy adults [file sj-docx-1-jop-10.1177_02698811251337397.docx]

**Supplementary Table 1.** Correlations between startle amplitude, habituation, and PPI.

| **Variables** | **1** | **2** | **3** | **4** | **5** | **6** | **7** | **8** | **9** | **10** | **11** | **12** | **13** | **14** |
| --- | --- | --- | --- | --- | --- | --- | --- | --- | --- | --- | --- | --- | --- | --- |
| **Morning (M) Session** | | | | | | | | | | | | | | |
| 1. Pulse-alone Amplitude - First Block | 1 | **0.708** | 0.217 | **0.826** | 0.260 | 0.262 | **0.452** | **0.610** | **0.645** | **-0.418** | **0.502** | 0.083 | -0.034 | **0.345** |
|  |  | **(<0.001)** | (0.210) | **(<0.001)** | (0.131) | (0.128) | **(0.006)** | **(<0.001)** | **(<0.001)** | **(0.012)** | **(0.002)** | (0.636) | (0.848) | **(0.042)** |
| 1. Pulse-alone Amplitude - Last Block |  | 1 | **-0.382** | **0.927** | 0.251 | 0.127 | **0.445** | **0.747** | **0.739** | **-0.400** | **0.761** | 0.045 | -0.091 | 0.318 |
|  |  |  | **(0.023)** | **(<0.001)** | (0.139) | (0.459) | **(0.007)** | **(<0.001)** | **(<0.001)** | **(0.016)** | **(<0.001)** | (0.793) | (0.596) | (0.059) |
| 1. Habituation: Reduction from First to Last Block |  |  | 1 | -0.118 | 0.056 | 0.115 | 0.248 | -0.135 | -0.058 | -0.030 | -0.191 | 0.060 | 0.066 | 0.122 |
|  |  |  |  | (0.501) | (0.747 | (0.509) | (0.152) | (0.438) | (0.743) | (0.863) | (0.273) | (0.730) | (0.708) | (0.487) |
| 1. Pulse-alone Amplitude - PPI Experiment |  |  |  | 1 | **0.352** | 0.208 | **0.579** | **0.774** | **0.798** | **-0.482** | **0.765** | 0.106 | -0.079 | **0.378** |
|  |  |  |  |  | **(0.035** | (0.224) | **(<0.001)** | **(<0.001)** | **(<0.001)** | **(0.003)** | **(<0.001)** | (0.539) | (0.647) | **(0.023)** |
| 1. PPI30 |  |  |  |  | 1 | **0.427** | **0.651** | 0.299 | 0.292 | -0.054 | 0.210 | -0.003 | 0.003 | 0.093 |
|  |  |  |  |  |  | **(0.009)** | **(<0.001)** | (0.077) | (0.084) | (0.753) | (0.218) | (0.984) | (0.985) | (0.589) |
| 1. PPI60 |  |  |  |  |  | 1 | **0.418** | -0.132 | -0.105 | 0.003 | -0.063 | 0.160 | **0.530** | **0.344** |
|  |  |  |  |  |  |  | **(0.011)** | (0.444) | (0.541) | (0.986) | (0.714) | (0.351) | **(<0.001)** | **(0.040)** |
| 1. PPI120 |  |  |  |  |  |  | 1 | **0.551** | **0.482** | -0.138 | **0.464** | -0.036 | 0.150 | **0.378** |
|  |  |  |  |  |  |  |  | **(<0.001)** | **(0.003)** | (0.422) | **(0.004)** | (0.835) | (0.382) | **(0.023)** |
| **Late afternoon (LA) Session** | | | | | | | | | | | | | | |
| 1. Pulse-alone Amplitude - First Block |  |  |  |  |  |  |  | 1 | **0.884** | -0.236 | **0.759** | 0.047 | -0.116 | **0.420** |
|  |  |  |  |  |  |  |  |  | **(<0.001)** | (0.166) | **(<0.001)** | (0.788) | (0.499) | **(0.011)** |
| 1. Pulse-alone Amplitude - Last Block |  |  |  |  |  |  |  |  | 1 | **-0.621** | **0.812** | 0.177 | -0.094 | **0.433** |
|  |  |  |  |  |  |  |  |  |  | **(<0.001)** | **(<0.001)** | (0.301) | (0.585) | **(0.008)** |
| 1. Habituation: Reduction from First to Last Block |  |  |  |  |  |  |  |  |  | 1 | **-0.540** | -0.261 | -0.036 | -0.233 |
|  |  |  |  |  |  |  |  |  |  |  | **(<0.001)** | (0.125) | (0.836) | (0.171) |
| 1. Pulse-alone Amplitude - PPI Experiment |  |  |  |  |  |  |  |  |  |  | 1 | 0.175 | 0.208 | **0.432** |
|  |  |  |  |  |  |  |  |  |  |  |  | (0.308) | (0.223) | **(0.009)** |
| 1. PPI30 |  |  |  |  |  |  |  |  |  |  |  | 1 | **0.345** | **0.574** |
|  |  |  |  |  |  |  |  |  |  |  |  |  | **(0.039)** | **(<0.001)** |
| 1. PPI60 |  |  |  |  |  |  |  |  |  |  |  |  | 1 | **0.497** |
|  |  |  |  |  |  |  |  |  |  |  |  |  |  | **(0.002)** |
| 1. PPI120 |  |  |  |  |  |  |  |  |  |  |  |  |  | 1 |

**Abbreviation:** PPI, Prepulse Inhibition
